# Indoor PM Pollution and Elevated Blood Pressure: Cardiovascular Impact of Indoor Biomass Burning

**DOI:** 10.1289/ehp.119-a442b

**Published:** 2011-10-01

**Authors:** Bob Weinhold

**Affiliations:** Bob Weinhold, MA, has covered environmental health issues for numerous outlets since 1996. He is a member of the Society of Environmental Journalists.

Elevated blood pressure leads to increased risk of cardiovascular disease, stroke, and kidney disease. Indoor air pollution, including exposure to fine particulate matter (PM_2.5_), has been hypothesized to contribute to elevated blood pressure, although little epidemiologic research has been conducted on this potential link. To investigate the issue further, a team of U.S., British, and Chinese researchers assessed the link between blood pressure and PM_2.5_ emitted during indoor burning of wood, coal, or crop residues used for heating and cooking [*EHP* 119(10):1390–1395; Baumgartner et al.]. These fuels and the poorly vented, inefficient stoves in which they are typically burned are a significant source of indoor air pollution exposure for almost half the world’s population.

The researchers evaluated the blood pressure of 280 Chinese women, aged 25–90, in conjunction with their personal exposure to PM_2.5_, as measured by a device the women wore or set nearby during two to six 24-hour periods over the course of a year. Blood pressure was measured immediately before and after each 24-hour PM_2.5_ measurement. The researchers were able to account for other factors that affect blood pressure, including age, education, height, weight, physical activity, salt intake, medication use, smoking, secondhand smoke exposure, caffeine consumption, pregnancy, medical history, air temperature, season, and socioeconomic status.

**Figure:**
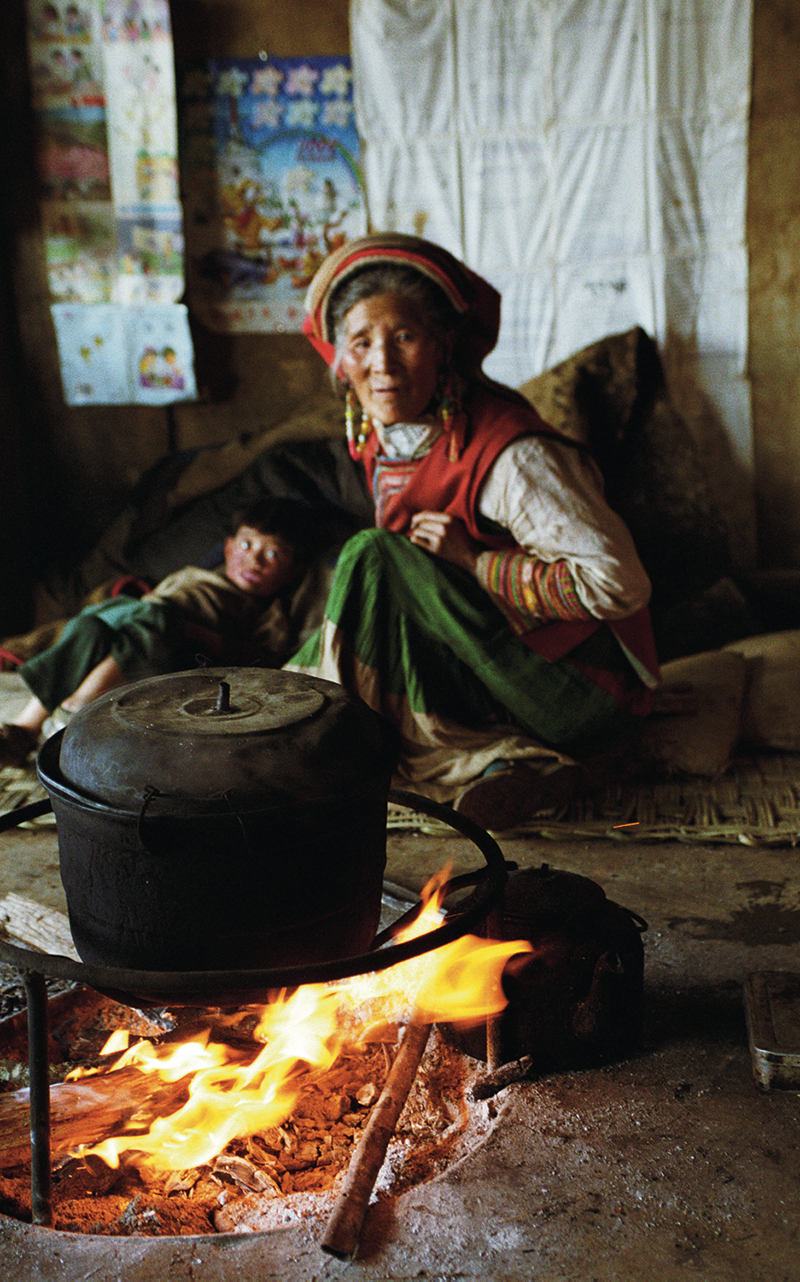
Indoor burning of traditional biomass fuels, as in this home in Yunnan Province, China, results in significant indoor PM_2.5_ exposure. © Boaz Rottem/Alamy

The 24-hour PM_2.5_ exposures ranged from 22 to 634 µg/m^3^ in winter (mean 117 µg/m^3^) and 9 to 492 µg/m^3^ in summer (mean 55 µg/m^3^); by comparison, the EPA 24-hour standard is 35 µg/m^3^. There was a significant association between increasing PM_2.5_ exposure and elevated blood pressure in women over age 50, with an increase in systolic blood pressure of 4.1 mmHg and an increase in diastolic blood pressure of 1.8 mmHg for each log-µg/m^3^ increase in PM_2.5_.

Few studies have investigated the link between chronic PM exposures and the development of overt hypertension. However, the authors estimate, based on previous reports, that reducing systolic blood pressure by 4 mmHg among Chinese women aged 50–59 could lead to an 18% decrease in coronary heart disease, a 22% decrease in stroke, and about 231,000 fewer premature deaths among Chinese women over age 50. They say such reductions could be accomplished if households switched from open fires and other inefficient cooking and heating devices to more efficient stoves (such as improved wood-burning cookstoves) and less polluting fuels (such as liquefied petroleum gas).

